# Supplementary material for: Integrated care in patients with atrial fibrillation- a predictive heterogeneous treatment effect analysis of the ALL-IN trial
Source: PLoS One. 2023 Oct 19;18(10):e0292586. doi: 10.1371/journal.pone.0292586 (PMC10586661; doi:10.1371/journal.pone.0292586)
Supplement: S1 Table — (DOCX) [file pone.0292586.s001.docx]

**S1 Table. Patient characteristics of study population for model development and the the ALL-IN study population**

|  | Population for model development (N=2355) | ALL-IN study population (N=1240) |
| --- | --- | --- |
| Median age (IQR) | 77.0 (16) | 77.0 (11) |
| Median CHA_2_DS_2_-VASC score (IQR) | 3.00 (3) | 3.00 (2) |
| Female sex | 1014 (43.1) | 613 (49.4) |
| Hypertension | 1411 (60.0% | 700 (56.5) |
| Heart failure | 432 (18.3) | 208 (16,8) |
| Diabetes | 529 (22.5) | 316 (25.5) |
| Prior stroke/TIA | 386 (16.4) | 179 (14.4) |
| Coronary artery disease | 628 (26.7) | 213 (17.2) |
| Prior myocardial infarction | 281 (11.9) | 86 (6.9) |
| Peripheral artery disease | 201 (8.5) | 84 (6.8) |
| Prior venous thromboembolism | 193 (8.2) | 55 (4.4) |
| Renal insufficiency | 349 (14.8) | 169 (13.6) |
| COPD | 352 (14.9) | 172 (13.9) |
| History of cancer | n.a. | 226 (18.2) |

*Data are numbers (percentage) unless stated otherwise. IQR: interquartile range. CHA_2_DS_2_-VASc: (Congestive heart failure, Hypertension, Age, Diabetes, prior Stroke, Vascular disease and Sex). TIA: transient ischemic attack. COPD: chronic obstructive pulmonary disease. n.a.: not available*
