# Supplementary material for: Integrated care in patients with atrial fibrillation- a predictive heterogeneous treatment effect analysis of the ALL-IN trial
Source: PLoS One. 2023 Oct 19;18(10):e0292586. doi: 10.1371/journal.pone.0292586 (PMC10586661; doi:10.1371/journal.pone.0292586)
Supplement: S2 Table — (DOCX) [file pone.0292586.s002.docx]

**S2 Table. Development and internal validation of the prediction model**

| Predictor | Regression coefficient | Standard error |
| --- | --- | --- |
| Age | 0.0814 | 0.0232 |
| Age’ | 0.0022 | 0.0183 |
| Sex | 0.0032 | 0.1306 |
| Hypertension | -0.1980 | 0.1308 |
| Diabetes | 0.4630 | 0.1388 |
| Stroke | 0.4083 | 0.1434 |
| Vascular disease | 0.1011 | 0.1368 |
| Heart failure | -0.0237 | 0.1482 |
|  | | |
| C-statistic after internal validation | 0.72 | 95% CI [0.69;0.75] |
| R squared | 0.093 |  |

*A cubic spline with 3 knots was used to account for non-linearity of the variable age. The variable age is therefore divided in 2 groups depicted as age, age’.*
