# Supplementary figures and images for: Integrated care in patients with atrial fibrillation- a predictive heterogeneous treatment effect analysis of the ALL-IN trial
Source: PLoS One. 2023 Oct 19;18(10):e0292586. doi: 10.1371/journal.pone.0292586 (PMC10586661; doi:10.1371/journal.pone.0292586)

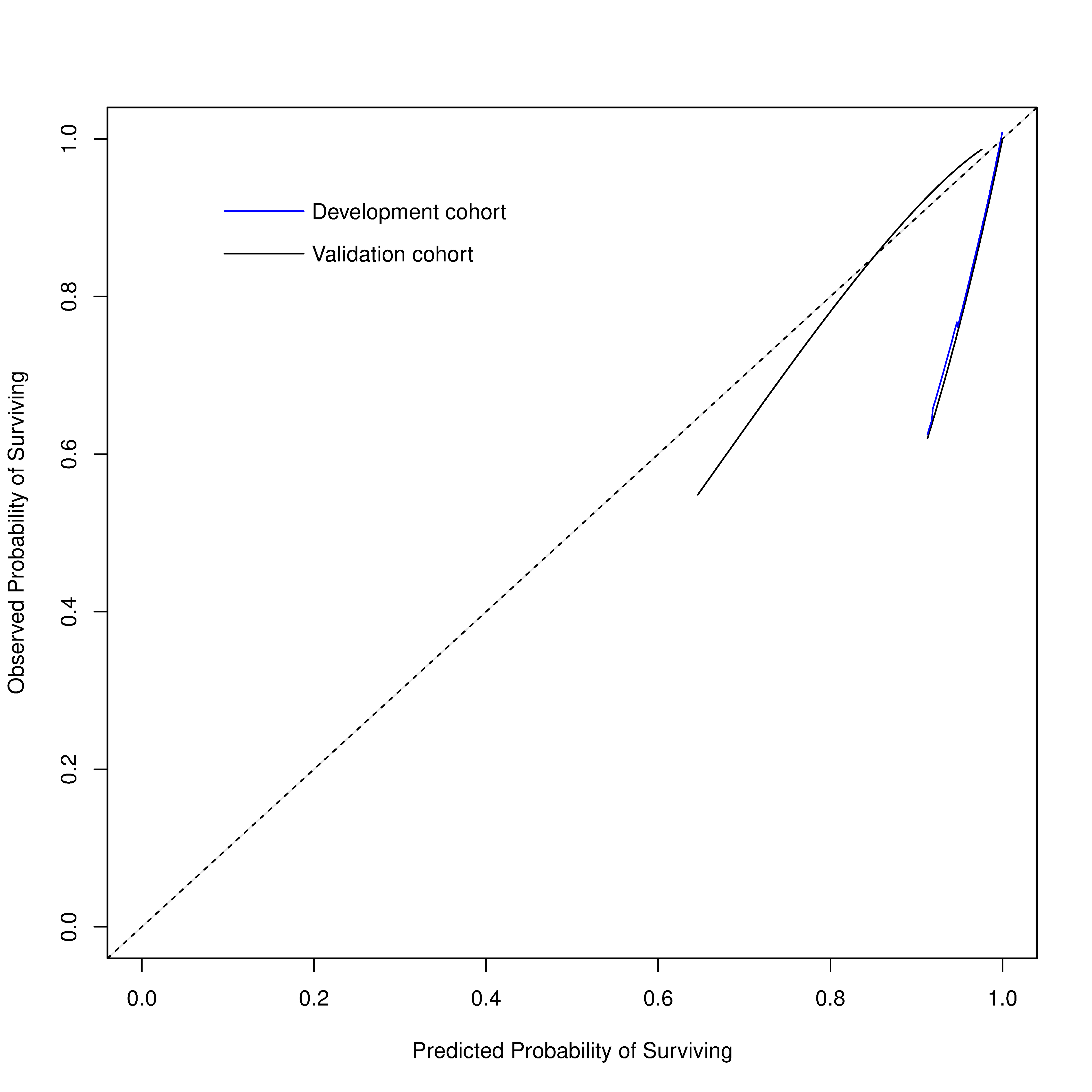

Supplement: S1 Fig — (TIF) [file pone.0292586.s003.tif]

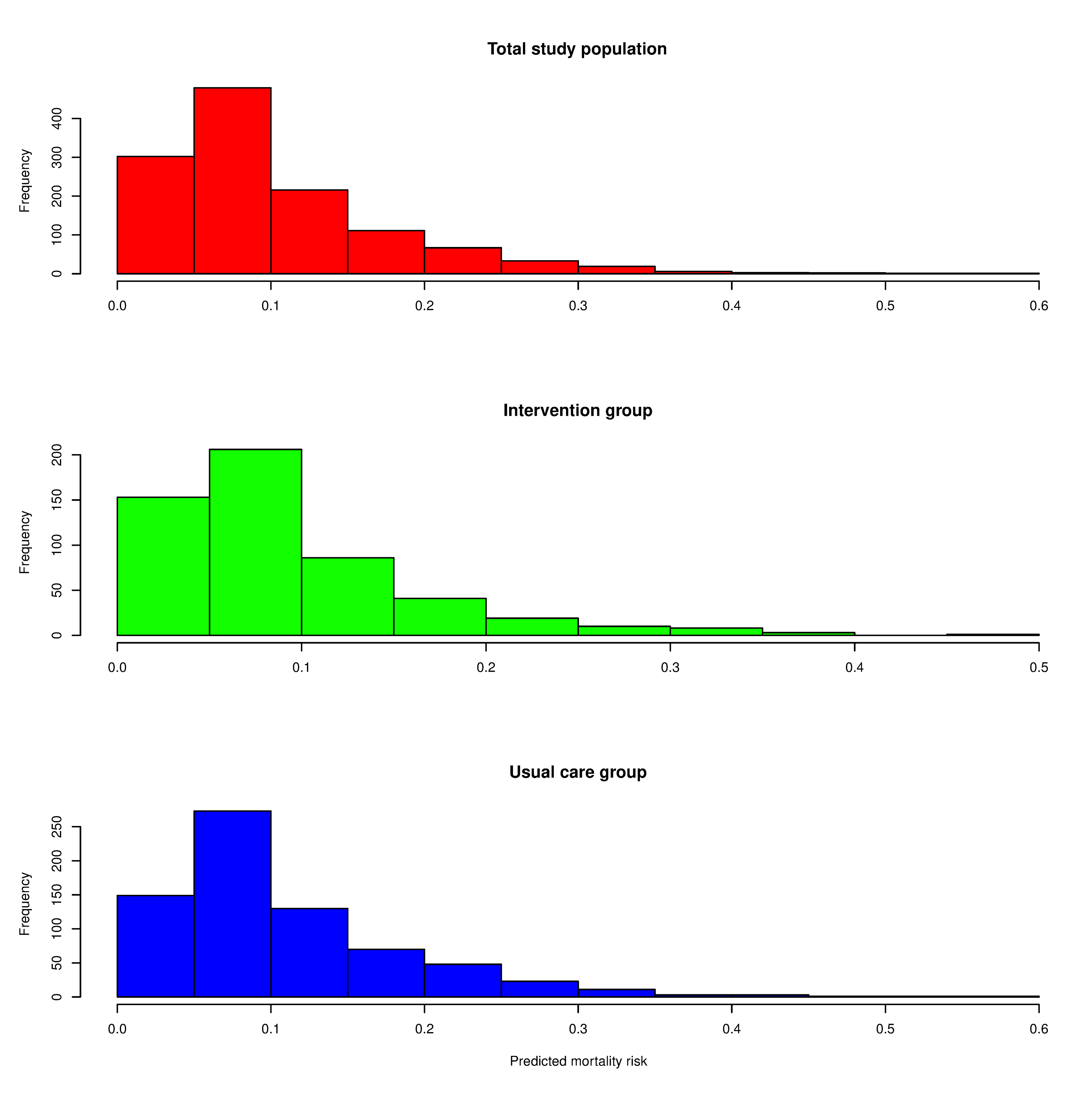

Supplement: S2 Fig — (TIF) [file pone.0292586.s004.tif]

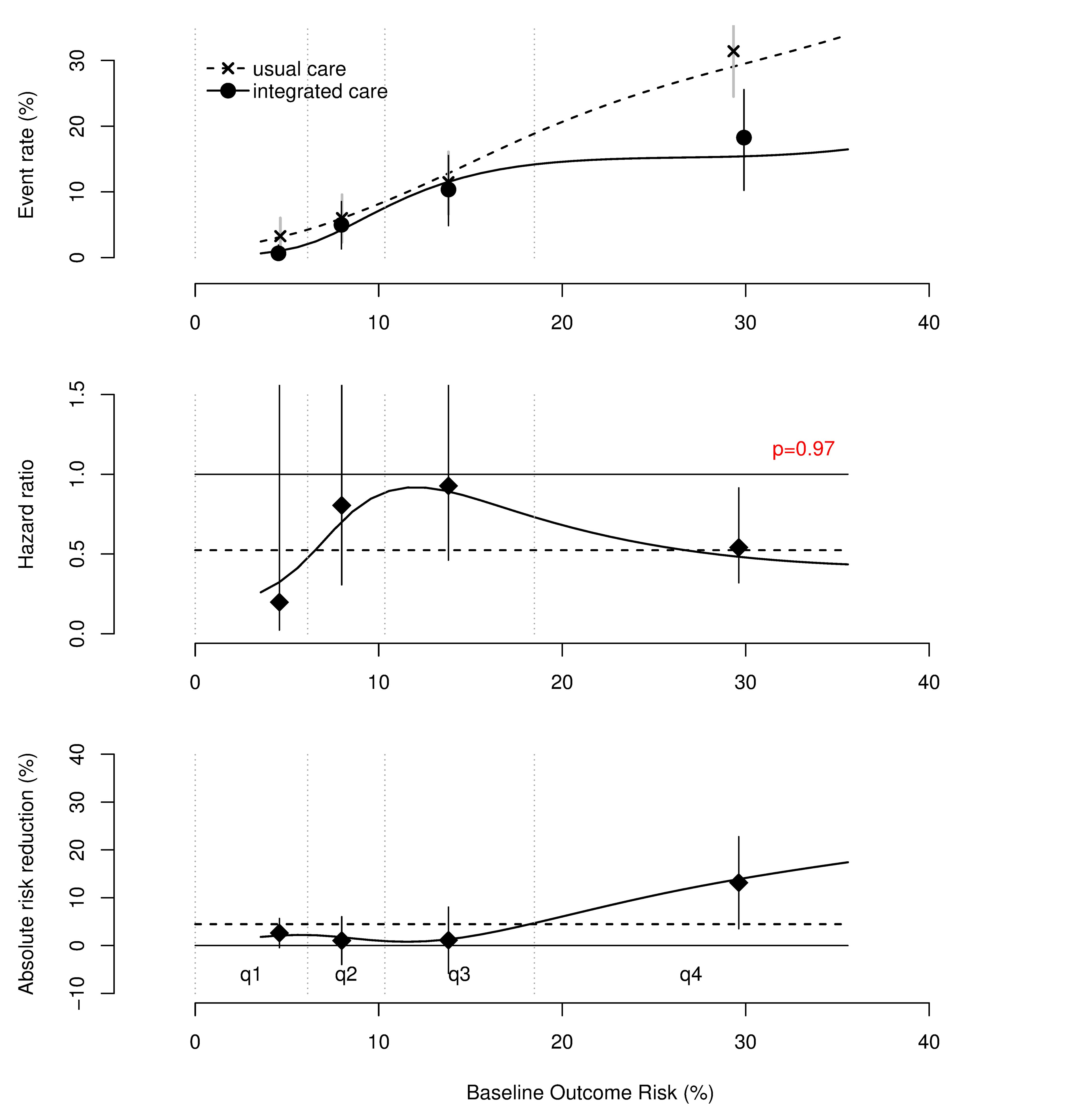

Supplement: S3 Fig — The event rate (top), the hazard ratios (middle), and the absolute risk reduction (bottom) are plotted as a function of the baseline outcome risk (i.e., predicted 2-year all-cause mortality risk). The intervention group (integrated AF care) is compared to usual care. The dashed line depicts the average effect (HR 0.55). q1, q2, q3 and q4 are four risk quarters. The vertical lines are 95% confidence intervals. (TIF) [file pone.0292586.s005.tif]

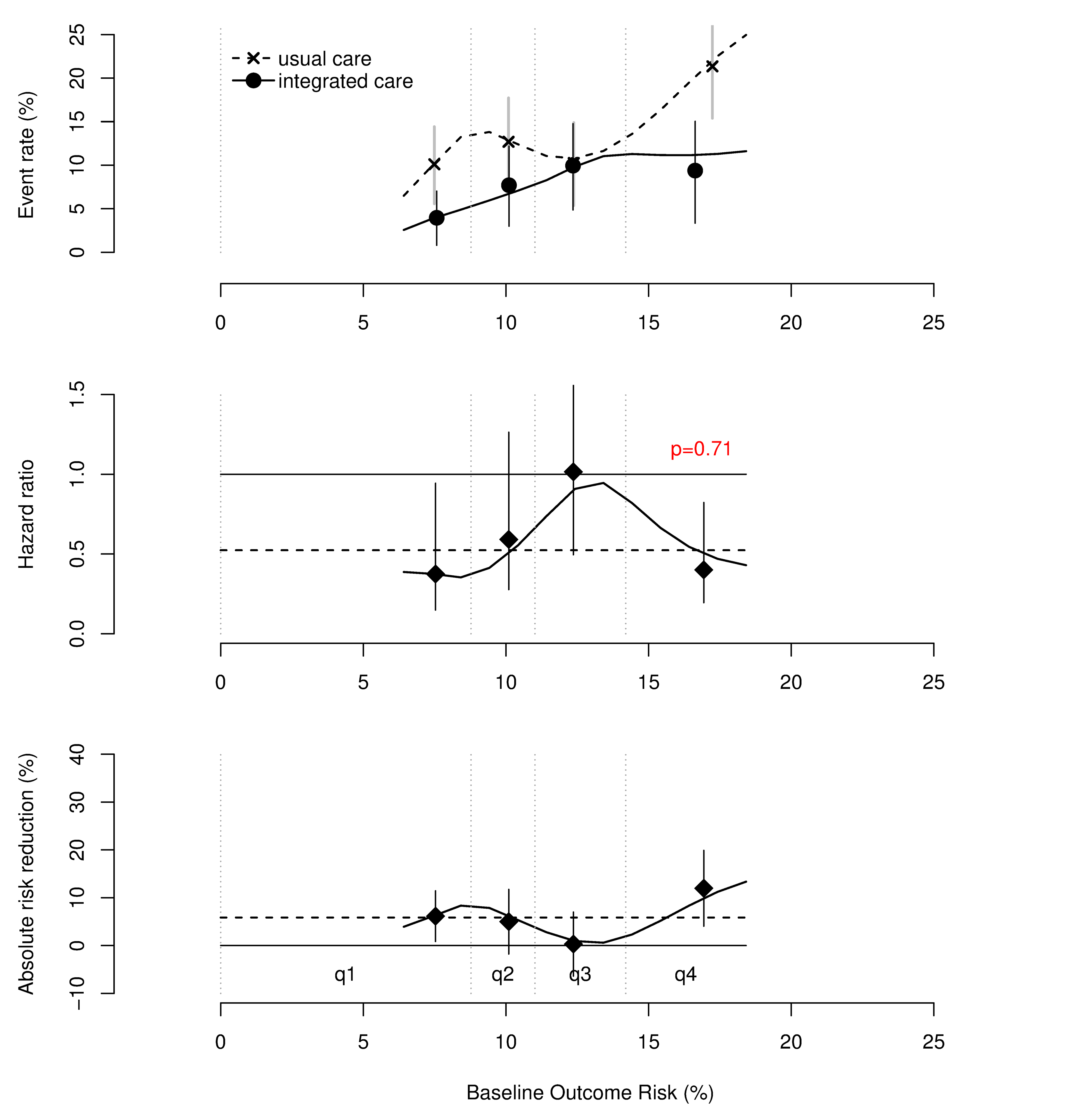

Supplement: S4 Fig — The event rate (top), the hazard ratios (middle), and the absolute risk reduction (bottom) are plotted as a function of the baseline outcome risk (i.e., predicted 2-year all-cause mortality risk). The intervention group (integrated AF care) is compared to usual care. The dashed line depicts the average effect (HR 0.55). q1, q2, q3 and q4 are four risk quarters. The vertical lines are 95% confidence intervals. (TIF) [file pone.0292586.s006.tif]
