# Supplementary material for: Integrated care in patients with atrial fibrillation- a predictive heterogeneous treatment effect analysis of the ALL-IN trial
Source: PLoS One. 2023 Oct 19;18(10):e0292586. doi: 10.1371/journal.pone.0292586 (PMC10586661; doi:10.1371/journal.pone.0292586)
Supplement: S1 Appendix — (DOCX) [file pone.0292586.s007.docx]

**Appendix**

**Integrated care in patients with atrial fibrillation- a predictive heterogeneous treatment effect analysis of the ALL-IN trial**

**Figure A1. Calibration plot of the internal validation of the prediction model**

**Figure A2. Calibration plot external validation of the prediction model**

**Figure A3. Distribution of predicted risk of all-cause mortality in total ALL-IN study population, intervention group and usual care group.**
