# Supplementary material for: Integrated care in patients with atrial fibrillation- a predictive heterogeneous treatment effect analysis of the ALL-IN trial
Source: PLoS One. 2023 Oct 19;18(10):e0292586. doi: 10.1371/journal.pone.0292586 (PMC10586661; doi:10.1371/journal.pone.0292586)
Supplement: Attachment — Submitted filename: 20230803_rebuttal_ALL-IN-HTE_PONE.docx [file pone.0292586.s008.docx]

August 24^th^, 2023

PONE-D-23-16112

*Integrated care in patients with atrial fibrillation- a predictive heterogeneous treatment effect analysis of the ALL-IN trial*

PLOS ONE

Dear editor,

Thank you for providing the opportunity to revise ort manuscript entitled “Integrated care in patients with atrial fibrillation- a predictive heterogeneous treatment effect analysis of the ALL-IN trial” for consideration for publication in PLOS ONE.

Please find below a point-by-point response to the editorial and reviewers’ comments.

**Journal requirements:**

**2. Thank you for stating the following in the Competing Interests section. (…)**

**Please confirm that this does not alter your adherence to all PLOS ONE policies on sharing data and materials, by including the following statement: "This does not alter our adherence to  PLOS ONE policies on sharing data and materials.”**

We included the aforementioned statement.

**3. We note that you have indicated that data from this study are available upon request. PLOS only allows data to be available upon request if there are legal or ethical restrictions on sharing data publicly. For more information on unacceptable data access restrictions, please see** [**http://journals.plos.org/plosone/s/data-availability#loc-unacceptable-data-access-restrictions**](http://journals.plos.org/plosone/s/data-availability#loc-unacceptable-data-access-restrictions)**.**

**In your revised cover letter, please address the following prompts:**

**a) If there are ethical or legal restrictions on sharing a de-identified data set, please explain them in detail (e.g., data contain potentially sensitive information, data are owned by a third-party organization, etc.) and who has imposed them (e.g., an ethics committee). Please also provide contact information for a data access committee, ethics committee, or other institutional body to which data requests may be sent.**

**b) If there are no restrictions, please upload the minimal anonymized data set necessary to replicate your study findings as either Supporting Information files or to a stable, public repository and provide us with the relevant URLs, DOIs, or accession numbers. For a list of acceptable repositories, please see** [**http://journals.plos.org/plosone/s/data-availability#loc-recommended-repositories**](http://journals.plos.org/plosone/s/data-availability#loc-recommended-repositories)**.**

**We will update your Data Availability statement on your behalf to reflect the information you provide.**

All relevant summary data are provided in the paper and its supplemental information. Part of the original data used in this study was collected during routine care and is formally owned by The Julius General Practitioners Network, JGPN, <https://portal.juliuscentrum.nl/research/nl-nl/cohortsandprojects/cohortsprojects/juliusgeneralpractitionersnetwork/jhn.aspx)>. Unfortunately, the owner of the data did not provide permission to make the data publicly available due to ethical and legal restrictions. The de-identified data are traceable to the participating centers who did not explicitly approve of raw data publication. However, all interested readers may request data without restriction from Dr. Sander van Doorn ([S.vanDoorn@umcutrecht.nl](mailto:S.vanDoorn@umcutrecht.nl)), the study coordinator of the JGPN.

**4. Your ethics statement should only appear in the Methods section of your manuscript. If your ethics statement is written in any section besides the Methods, please delete it from any other section.**

We restricted the ethics statement to the Methods section.

**5. Please include captions for your Supporting Information files at the end of your manuscript, and update any in-text citations to match accordingly. Please see our Supporting Information guidelines for more information:** [**http://journals.plos.org/plosone/s/supporting-information**](http://journals.plos.org/plosone/s/supporting-information)**.**

We formatted the Supporting Information, provided caption in the manuscript, and updated all in-text citations as per the instructions.

**Reviewer #1**

**I have reviewed the manuscript entitled “Integrated care in patients with atrial fibrillation- a predictive heterogeneous treatment effect analysis of the ALL-IN trial" by Trinks-Roerdink et al.
This is a nice and interesting study demonstrating that AF patients at higher risk of mortality are actually those who benefit more from integrated and holistic care.
The manuscript is clear and well-written. From a methodological point of view, I do not see major issues or concerns.
However, I do not understand well the rationale of the developed a new prediction model for all-cause mortality. Indeed, it is not declared as one of the objectives of the study, and the explanation of this result in the Results section is very brief. In my opinion, it does not add too much to the study and probably deserves another paper where the authors should give more details.**

We thank the reviewer for their interest in our study. Indeed, developing a new prediction model for all-cause mortality was not the objective of our study, but merely a method to provide patients with a baseline risk in order to assess the heterogeneity in the effect of integrated care. While we recognize the importance of complete and transparent reporting of the development of any new prediction model (as advocated in the TRIPOD statement),(1) we adhered to the PATH statement for HTE analyses.
We now elaborate on this in the Strengths and Limitation paragraph in the Discussion section (page 14, line 317 onwards):

*“It was not our primary aim to develop a model for formal use to predict mortality in future clinical practice, and we did not consider predictors beyond the clinical variables from the CHA_2_DS_2_-VASc score. Such additional variables beyond those included in the extensive model in the sensitivity analysis may predict mortality but were not (uniformly) available in both datasets. Nonetheless, the predictive performance of the model was good.”*

and made some additional minor changes throughout, with track changes.

**Minor comments:
CHADS-VASc should be spelled out as CHA2DS2-VASc in the abstract, discussion, and conclusion.**

We deliberately chose to formulate the predictors as derived from CHADS-VASc as we did *not* apply any scoring (with double points for Age ≥75 and previous stroke) but used unrounded coefficients instead. Prompted by this valuable comments, we rephrased this to “clinical variables from the CHA2DS2-VASc score” to avoid confusion.

**Reviewer #2:**

**The manuscript presented reports on a very interesting analysis regarding the impact of an integrated care strategy in reducing risk of all-cause death, according to predicted baseline risk in an HTE analysis.
The study surely poses an interesting question, focusing on a relevant clinical problem. Indeed, in this era in which limited resources are available (and personalised medicine is an aim), could be needed to identify more carefully patients to be treated according to certain standards, in order to maximize the effectiveness of clinical strategies.
The results showed are extremely interesting, since underlining the fact that patients with a higher predicted risk can gather the larger reduction in risk when treated with an integrated care approach is extremely important to facilitate the implementation of such clinical strategies.
Notwithstanding, I thing some issues are really important to be managed in the manuscript:**

**- I'm a bit concerned about the model used to evaluated the baseline predicted risk. The authors explained that a model was derivated from an external cohort, using the same factors part of the CHA2DS2-VASc score (but not the score itself, since they argue that was not originally validated for all-cause death) and then applied to the study cohort, to avoid biases related to the fact that the same factors could influence the choice of treatment. First of all, I don't see the same danger of bias related to the confounding-by-indication bias. If we consider that the original study was a cluster randomized trial, all the patients in each cluster received the same treatment approach, hence none of the clinical factors should have influenced it.**

We thank the reviewer for recognizing the clinical problem our study aims to address. As for the development of the prediction model in an external dataset, we agree that confounding-by-indication will not be a concern in the ALL-IN trial due to its (cluster-)randomized design. However, as advocated in the PATH statement, a prediction model for HTE analysis should ideally be developed in an external dataset to avoid any overfitting. Hence our use of data from a different study in a different timeframe, with external validation in the ALL-IN study.

**Second, the cohort used to derive the model is almost ten years old and, very likely, including patients completely different from those included in this current study.**

This is a very valuable remark. We now included a table comparing the patient characteristics in the derivation cohort with those in the study cohort as Supporting Information S1.

**Third, if we should use a different model, I would not use only those few clinical factors, but would enlarge the list including other important factors influencing the risk of death, such as CKD, COPD, Liver Disease; all these conditions have shown a direct independent influence on the risk of death in AF patients.**

We completely agree with the reviewer that many more factors can predict mortality in AF patients. Importantly, though, we did not intent to develop a model for formal use in prediction mortality in daily practice and, despite using a limited set of predictors, our model already had good predictive performance (c-statistic 0.72 upon external validation).

We now elaborate more on this as a limitation in the Discussion section (page 14, line 317 onwards):

*“It was not our primary aim to develop a model for formal use to predict mortality in future clinical practice, and we did not consider predictors beyond the clinical variables from the CHA_2_DS_2_-VASc score. Such additional variables beyond those included in the extensive model in the sensitivity analysis may predict mortality but were not (uniformly) available in both datasets. Nonetheless, the predictive performance of the model was good.”*

Nonetheless, prompted by this comment we now include a sensitivity analysis using an extended model to predict mortality, with chronic kidney disease, COPD and liver disease and describe so in the Methods and Results section (page 7 line 166):

*“We performed two sensitivity analyses. First, we repeated the HTE analysis using an extended prediction model additionally including chronic kidney disease, COPD and liver disease. Second, we used the CHA_2_DS_2_-VASc score as a continuous numeric univariate predictor of mortality.”*

And (page 10, line 221):

*“Repeating the analyses using the extended model additionally including chronic kidney disease, COPD and liver disease or the CHA_2_DS_2_-VASc score as a univariate predictor did not substantially change the results, see Fig in S5A Fig and Fig in S5B Fig .”*

**Fourth, despite not being originally validated for all-cause death, there are solid proofs that CHA2DS2-VASc is able to predict all-cause death occurrence (doi: 10.1177/2047487318817662). I would advise the authors, if they want to keep the current approach with the developed risk model (nonetheless I would consider whether to change the approach and develop the risk model in the current cohort, to then validate it externally), to show the baseline characteristics of the patients in the derivation cohort, at least to compare indirectly with the current cohort.**

We refer to our answer above and to Supporting Information S1 with a comparison of the patient characteristics in the derivation cohort with those in the study cohort.

**They would maybe try to create a larger model, as a sensitivity analysis, including more clinical factors. Also, I would advise to perform the same analysis using the actual CHA2DS2-VASc score, considering the fact that is widely used in the clinical practice; this would allow to put their results more easily in the daily clinical practice. Such results would strenghten their main one.**

The reviewer makes a valuable suggestion. Notably, the aim of our study was not to develop a formal model for predicting mortality in clinical practice and additionally stress so in de Discussion section (see above). However, to this extent it is certainly valuable to use the original CHA2DS2-VASc score for stratifying patients on baseline mortality risk. Prompted by this novel idea, we performed this additional analyses and report this in the Methods and Results section and refer to the changes above.

**Another issue related to the predicted risk model is related to the calibration. The two plots (which actually I will incorporate in the main paper, instead avoiding Table 2 which is only little informative) looks like having completely different scales and then are impossible to be compared even visually, but even to make any assumptions. Indeed, by looking at the Supplementary Figure 2, it seems like the calibration is not so good in the lower risk group, but being the axis truncated at 0.6 is difficult to be completely sure about what we.
The authors should put both the curves on the same figure, not truncate any axes and use the same scale, including the figure in the main paper (also with a clear legend, that is currently missing). They should then comment and build up more on this point. Indeed, considering that the most of the patients have a low risk, is essential that the model perform at least acceptably in such patients.**
We thank the reviewer for these suggestions. We now provide a Figure consisting of a calibration plot including both curves, with untruncated axes, and a clear legend. Of note, the curves show the predicted and observed probability of *survival* and thus most patients are at *low* mortality risk (upper right part of the plot). It is at the low risk of mortality where calibration is best. We now elaborate on this in de Results section (page 9, line 201):

*“See Figure in S3 Figure for the calibration plots in the study population for model development and the ALL-IN population. In both, most patients had a high probability of survival (i.e. a low risk of mortality) which was mostly overestimated by the prediction model. Calibration was best mainly for the higher probabilities of survival (i.e. lower risk of mortality), for which we have the most observations.”*

We deliberately chose to show the calibration plots in the supplemental material instead of the main paper, as we did not want to put too much emphasis on the model development itself to avoid confusion regarding the aim of the study. Consequently, prompted by the reviewer comments, we additionally propose to move Table 2 to the Supporting Information.

**- I think the authors could build up more about the impact of their results, regarding the issue of multimorbidity and frailty, which are very common in AF patients (DOI: 10.1016/j.lanepe.2022.100386, 10.1016/j.arr.2022.101652). How would their finding impact in the clinical management of such patients?**

We wholeheartedly agree with the reviewer that AF and frailty are unequivocally intertwined. Many AF patients indeed are frail, due to multimorbidity and polypharmacy but in fact due to many other aspects of the ‘frailty syndrome’ as well. We believe our results show that it is in particular in these patients that integrated care provides most benefit. We now elaborate on this in the ‘Discussion’ section of our manuscript and added also the reference as suggested by the reviewer (page 12, line 284):

*“With increasing age, there is an accumulation of risk factors for all-cause mortality, due to ageing but also due to interacting comorbidities. These comorbidities along with polypharmacy, high biological vulnerability, dependency on significant others and a reduced capacity to resist stressors, together create frailty that so often is found in AF patients. In fact, addressing all these components of frailty at once are the exact merits of integrated AF care. The results of our analysis of heterogeneity in treatment effect show that the higher the burden of comorbidities – and thus possibly the higher the frailty – the greater the effect of integrated AF care. This exemplifies the importance of addressing the ‘C’ component (detection and management of comorbidities) in the ABC approach.”*

**Additional references**

Moons, K. G., Altman, D. G., Reitsma, J. B., Ioannidis, J. P., Macaskill, P., Steyerberg, E. W., Vickers, A. J., Ransohoff, D. F., & Collins, G. S. (2015). Transparent Reporting of a multivariable prediction model for Individual Prognosis or Diagnosis (TRIPOD): explanation and elaboration. *Ann Intern Med*, *162*(1), W1-73. <https://doi.org/10.7326/M14-0698>
